# Cysteine boosters the evolutionary adaptation to CoCl_2_ mimicked hypoxia conditions, favouring carboplatin resistance in ovarian cancer

**DOI:** 10.1186/s12862-018-1214-1

**Published:** 2018-06-19

**Authors:** Sofia C. Nunes, Filipa Lopes-Coelho, Sofia Gouveia-Fernandes, Cristiano Ramos, Sofia A. Pereira, Jacinta Serpa

**Affiliations:** 1 Centro de Estudos de Doenças Crónicas (CEDOC), NOVA Medical School/Faculdade de Ciências Médicas, Universidade Nova de Lisboa, Campo Mártires da Pátria 130, 1169-056 Lisbon, Portugal; 2 Unidade de Investigação em Patobiologia Molecular do Instituto Português de Oncologia de Lisboa Francisco Gentil (IPOLFG), Rua Prof Lima Basto, 1099-023 Lisbon, Portugal

**Keywords:** Metabolic selection, Evolutionary trade-off, Ovarian cancer, Cysteine, Hypoxia, Chemoresistance

## Abstract

**Background:**

Ovarian cancer is the second most common gynaecologic malignancy and the most common cause of death from gynaecologic cancer, especially due to diagnosis at an advanced stage, when a cure is rare. As ovarian tumour grows, cancer cells are exposed to regions of hypoxia. Hypoxia is known to be partially responsible for tumour progression, metastasis and resistance to therapies. These suggest that hypoxia entails a selective pressure in which the adapted cells not only have a fitness increase under the selective environment, but also in non-selective adverse environments. In here, we used two different ovarian cancer cell lines – serous carcinoma (OVCAR3) and clear cell carcinoma (ES2) – in order to address the effect of cancer cells selection under normoxia and hypoxia mimicked by cobalt chloride on the evolutionary outcome of cancer cells.

**Results:**

Our results showed that the adaptation to normoxia and CoCl_2_ mimicked hypoxia leads cells to display opposite strategies. Whereas cells adapted to CoCl_2_ mimicked hypoxia conditions tend to proliferate less but present increased survival in adverse environments, cells adapted to normoxia proliferate rapidly but at the cost of increased mortality in adverse environments. Moreover, results suggest that cysteine allows a quicker response and adaptation to hypoxic conditions that, in turn, are capable of driving chemoresistance.

**Conclusions:**

We showed that cysteine impacts the adaptation of cancer cells to a CoCl_2_ mimicked hypoxic environment thus contributing for hypoxia-drived platinum-based chemotherapeutic agents’ resistance, allowing the selection of more aggressive phenotypes. These observations support a role of cysteine in cancer progression, recurrence and chemoresistance.

**Electronic supplementary material:**

The online version of this article (10.1186/s12862-018-1214-1) contains supplementary material, which is available to authorized users.

## Background

Ovarian cancer is the major cause of death from gynaecologic disease and the second most common gynaecologic malignancy worldwide [1, 2], especially due to late diagnosis and resistance to therapy [3]. Epithelial ovarian carcinoma (EOC) includes most malignant ovarian neoplasms [4], being the high-grade ovarian serous carcinoma (OSC) the most prevalent histological type [3] with diagnosis at an advanced stage in approximately 70% of patients [5]. In contrast, ovarian clear cell carcinoma (OCCC), is a rather uncommon histological type of ovarian cancer that is frequently diagnosed at an initial stage [6]. However, tumours present markedly different clinical behaviours compared to other epithelial ovarian cancers presenting, generally, poor prognosis given chemoresistance to conventional platinum-drugs and taxane-based chemotherapy [6]. The standard care for ovarian cancer is a combination of surgery and paclitaxel–carboplatin therapy [7]. However, despite initial response, there is a recurrence of the disease in over 85% of advanced ovarian cancer patients [8]. Usually, OSC shows an initial response to platinum based therapy with further progression to resistance [9] while OCCC is intrinsically resistant to platinum salts [6, 10, 11].

Serpa and Dias have suggested that the metabolic remodelling is determinant for tumour progression. They have proposed a model in which the selective pressure of the microenvironment involving metabolic pathways switching induces cell death in non-adapted cells and positively selects those cells with growth advantage, increased invasion and altered adhesiveness. This allows local and angio (vascular) invasion, ultimately leading to cancer progression and distant metastasis [12]. Soon after this report, Hanahan and Weinberg had also included reprogramming of energy metabolism as an emerging hallmark of cancer [13].

As a solid tumour grows, cancer cells are exposed to regions of hypoxia. The effects of intermittent hypoxia on cancer biology have been related to the aberrant blood circulation observed in solid tumours. This results in recurrent intra-tumoral episodic hypoxia and assaults metabolically less privileged cell niches. These studies showed that hypoxia is partially responsible for tumour progression, metastasis and resistance to therapies [14–17]. This evidence supports that hypoxia entails a selective pressure in which the adapted cells not only have a fitness increase under the selective environment, but also in non-selective adverse environments. Moreover, Cutter et al. [18] have recently reported that ovarian cancer cell lines subjected to hypoxia are more invasive, have a migratory ability and display a transformed epithelial–mesenchymal transition (EMT) phenotype. Hence, ovarian cancer is a valuable model to address the metabolic evolution driven by hypoxia.

The contribution of cysteine on cancer cells survival has been explored mainly due to hydrogen sulphide (H_2_S) generation [19–24] and as a precursor of the antioxidant glutathione (GSH) [25–27]. We and others showed that increased levels of cytoplasmic thiol-containing species, especially glutathione or metallothioneins are associated with resistance to platinum-based chemotherapy [25, 28, 29]. Our group also showed that different ovarian cancer histological types had different metabolic outcomes concerning thiols and chemoresistance [25]. Under normoxic conditions, the OCCC cells were more resistant to carboplatin than OSC cells and the inhibition of GSH production by buthionine sulphoximine (BSO) sensitized OCCC cells to carboplatin, in vitro and in vivo [25]. Those results suggest that the ability to metabolize thiols by cancer cells is directly linked to poorer disease outcome.

In this study, we used two different cancer cell lines derived from two different histological types of ovarian cancer (OCCC and OSC) and addressed the effect of cells selection under normoxia and hypoxia, mimicked by cobalt chloride (CoCl_2_), on the evolutionary outcome of cancer cells. Cobalt is known as a hypoxia mimicking agent both in vivo [30] and in cell culture [31–33]. Cobalt was shown to alter several systemic mechanisms related to hypoxia [31–33], namely the stabilization of hypoxia inducible factor alpha (HIF-α), thus preventing its degradation [34]. Chemically, CoCl_2_ reacts with oxygen impairing its dissolution and oxygenation of aqueous solutions [35], being a way of inducing unavailability of oxygen in culture media.

Herein, we hypothesised that selection under CoCl_2_ mimicked hypoxia and normoxia leads cells to display different evolutionary outcomes, predicting that hypoxia selected cells would be more resistant to carboplatin than normoxia selected cells. Moreover, we hypothesised that selection under hypoxia is linked to a higher bioavailability of cysteine, resulting in a poorer evolutionary outcome.

## Methods

### Cell culture

Cell lines from OCCC (ES2; CRL-1978) and OSC (OVCAR-3; HTB-161) were obtained from American Type Culture Collection (ATCC). Cells were maintained at 37 °C in a humidified 5% CO_2_ atmosphere. Cells were cultured in DMEM (41965–039, Gibco, Life Technologies) containing 4.5 g/L of D-glucose and 0.58 g/L of L-glutamine supplemented with 1% fetal bovine serum (FBS S 0615, Merck), 1% antibiotic-antimycotic (AA) (P06–07300, PAN Biotech).

Prior to any experiment, cells were synchronized under starvation (culture medium without FBS) for 8 h at 37 °C and 5% CO_2_.

After 24 h of conditions exposure, the medium was changed and fresh conditions were added, with the exception of proliferation curve and cell cycle analysis in which the medium was not changed.

### Immunoflorescense

Cells (4× 10^4^ cells/well) were seeded in 8-well Tek chamber slides (Thermo scientific 177,402) coated with Poly-L-Lysine (Biochrom AG L 7240) and cultured either in control condition or exposed to 100 μM CoCl_2_. Cells were collected after 8 h of conditions and then cells were fixed with 2% paraformaldehyde for 15 min at 4 °C and permeabilized with PBS-BSA 0.1% with 0.1% Triton X-100 for 30 min. Cells were incubated with primary antibody (anti-HIF-1α; anti-mouse antibody from BD Biosciences 610,959) overnight at 4 °C (diluted in PBA-BSA 0.1%, 1:100). Secondary antibody was the anti-mouse Alexa Fluor® 488 (1:1000 in PBA-BSA 0.1%. Antibody from Jackson ImmunoResearch Laboratories 115–545-003), 2 h at room temperature. The slides were mounted in VECTASHIELD media with DAPI (4′-6-diamidino-2-phenylindole) (Vector Labs) and examined by standard fluorescence microscopy using a Zeiss Imajer.Z1 AX10 microscope. Images were acquired and processed with CytoVision software.

### Cell lines selection

ES2 and OVCAR3 cells (1 × 10^6^ cells) were cultured in 25cm^2^ tissue culture flasks and selected under normoxia and under hypoxia mimicked with 100μM CoCl_2._ After reaching confluency (≈2 × 10^6^ cells) cells were trypsinised and cultured in 75cm^2^ tissue culture flasks, under selective conditions (hypoxia mimicked with 100μM CoCl_2_). Every 48 h, cells undergone passaging if confluency reached ~ 80% (~ 7.5 × 10^6^) or culture media was only changed if this confluency was not achieved. As the proliferation and survival rates were different between cell lines, ES2 and OVCAR3 were selected for 63 and 84 days, respectively. After the period of selection, cells were expanded in baseline culture conditions to reach the number of cells needed to performed all the assays. Cobalt is a hypoxia mimicking agent commonly used in both in vivo [30] and in vitro [31–33] studies. CoCl_2_ reacts with oxygen avoiding its dissolution and oxygenation of aqueous solutions [35], being a way of impairing the availability of oxygen in culture media. However, from now on the condition will be designated as CoCl_2_ mimicked hypoxia condition. Within each cell line, selection in normoxia and CoCl_2_ mimicked hypoxia was performed simultaneously. Ancestral cell line was cultured in base line conditions.

Table 1 presents the selection and culture conditions for ES2 and OVCAR3 cell lines.

**Table 1:** Ovarian cancer cell lines, selection and culture conditions

Cell line- histological type	Cell line code	Selection condition	Culture condition
Ovarian clear cell carcinoma (OCCC; ES2)	ES2-AN	Non selected ancestral cell line	Normoxia
	ES2-ANC	Non selected ancestral cell line	Normoxia + cysteine
	ES2-AH	Non selected ancestral cell line	Hypoxia
	ES2-AHC	Non selected ancestral cell line	Hypoxia + cysteine
	ES2-NN	Selected under Normoxia	Normoxia
	ES2-NNC	Selected under Normoxia	Normoxia + cysteine
	ES2-NH	Selected under Normoxia	Hypoxia
	ES2-NHC	Selected under Normoxia	Hypoxia + cysteine
	ES2-HN	Selected under Hypoxia	Normoxia
	ES2-HNC	Selected under Hypoxia	Normoxia + cysteine
	ES2-HH	Selected under Hypoxia	Hypoxia
	ES2-HHC	Selected under Hypoxia	Hypoxia + cysteine
Ovarian serous carcinoma (OSC; OVCAR3)	OVCAR3-AN	Non selected ancestral cell line	Normoxia
	OVCAR3-ANC	Non selected ancestral cell line	Normoxia + cysteine
	OVCAR3-AH	Non selected ancestral cell line	Hypoxia
	OVCAR3-AHC	Non selected ancestral cell line	Hypoxia + cysteine
	OVCAR3-NN	Selected under Normoxia	Normoxia
	OVCAR3-NNC	Selected under Normoxia	Normoxia + cysteine
	OVCAR3-NH	Selected under Normoxia	Hypoxia
	OVCAR3-NHC	Selected under Normoxia	Hypoxia + cysteine
	OVCAR3-HN	Selected under Hypoxia	Normoxia
	OVCAR3-HNC	Selected under Hypoxia	Normoxia + cysteine
	OVCAR3-HH	Selected under Hypoxia	Hypoxia
	OVCAR3-HHC	Selected under Hypoxia	Hypoxia + cysteine

### Proliferation curve assay

Cells selected under normoxia and CoCl_2_ mimicked hypoxia (5 × 10^4^cells/well), were seeded in 24-well plates and cultured either in normoxia or exposed to 100μM CoCl_2_. Cells were collected after 16 h, 32 h and 48 h of conditions. Cells were trypsinized and resuspended in 200 μL of PBS 1×. A total of 15 μL were collected and 5 μL of trypan blue were added. Cells were immediately counted. The remnant cells were used to cell cycle analysis. This assay was performed with 63 days of selection for ES2 cells and 35 days of selection for OVCAR3 cells.

### Cell cycle assay

Cells were harvested by centrifugation at 1200 rpm for 5 min and cells were fixed with 70% ethanol at 4 °C. Cells were then centrifuged at 1200 rpm for 5 min, followed by the supernatant discharge. Cells were incubated with 100 μL of propidium iodide (PI) solution (50 μg/ml PI, 0.1 mg/ml RNase A, 0.05% Triton X-100) for 40 min at 37 °C. After the incubation period, cells were washed with PBS 1×, centrifuged at 1500 rpm for 10 min at 4 °C and the supernatant was discarded. Cell pellets were suspended in 200 μL of PBS-BSA 0.1%. The acquisition was performed in a FACScalibur (Becton Dickinson). Data were analysed with FlowJo software (www.flowjo.com).

### Cell death assay

Cells selected under normoxia and CoCl_2_ mimicked hypoxia (2 × 10^5^ cells/well) were seeded in 12-well plates and cultured under normoxia and exposed to 400μM L-cysteine and/or 100μM CoCl_2_. In addition, cells were exposed to the previous conditions combined with carboplatin 25 μg/mL. Cells were collected after 48 h of tested conditions. For the analysis of the response dynamics to carboplatin, the cells were collected after 16 h, 24 h and 48 h of conditions. The ancestral (not selected) cell lines were also tested. Half of the cells were used to cell death analysis and the other half was used for ROS quantification. This assay was performed with 43 days of selection for ES2 cells and 84 days of selection for OVCAR3 cells.

Cells were harvested by centrifugation at 1200 rpm for 3 min, cells were incubated with 1 μL annexin V-Alexa Fluor® 488 (640,906, BioLegend) in 100 μL annexin V binding buffer 1× (10 mM HEPES (pH 7.4), 140 mM sodium chloride (NaCl), 2.5 mM calcium chloride (CaCl_2_)) and incubated at room temperature and in the dark for 15 min. After incubation, samples were rinsed with 0.1% (*w*/*v*) BSA (A9647, Sigma) in PBS 1× and centrifuged at 1200 rpm for 3 min. Cells were suspended in 200 μL of annexin V binding buffer 1× and 5 μL Propidium Iodide (PI; 50 μg/mL). Acquisition was performed with a FACScalibur (Becton Dickinson). Data were analysed with FlowJo software (www.flowjo.com).

### ROS quantification assay

Cells selected under normoxia and CoCl_2_ mimicked hypoxia (2 × 10^5^ cells/well) were seeded in 12-well plates and cultured in control condition and exposed to 400μM L-cysteine and/or 100μM cobalt chloride and/or carboplatin 25 μg/mL. Cells were collected after 48 h of tested conditions. The ancestral cell lines were also tested. This assay was performed with 43 days of selection for ES2 cells and 84 days of selection for OVCAR3 cells.

Cells were incubated for 15 min 37 °C with 2′, 7′-Dichlorofluorescin diacetate (D6883, Sigma) in a final concentration of 10 μM. The acquisition was performed with FACScalibur (Becton Dickinson). Data were analysed with FlowJo software (www.flowjo.com).

### Statistical analysis

Data are presented as the mean ± SD and all the graphics were done using the PRISM software package (PRISM 6.0 for Mac OS X; GraphPad software, USA, 2013). Assays were performed with 3 replicates per treatment. For comparisons of two groups, two-tailed independent-samples T-test was used. For comparison of more than two groups, One-way analysis of variance (ANOVA) with Tukey’s multiple-comparisons post hoc test was used. To assess the existence of a linear relationship between two variables, two-tailed Pearson correlation was used. Statistical significance was established as *p* < 0.05. All statistical analyses were performed using the IBM Corp. Released 2013. IBM SPSS Statistics for Macintosh, Version 22.0. Armonk, NY: IBM Corp. software.

## Results

### Adaptation to normoxia (N) confers a highly proliferative ability to ES2 cells

**Fig. 1:**
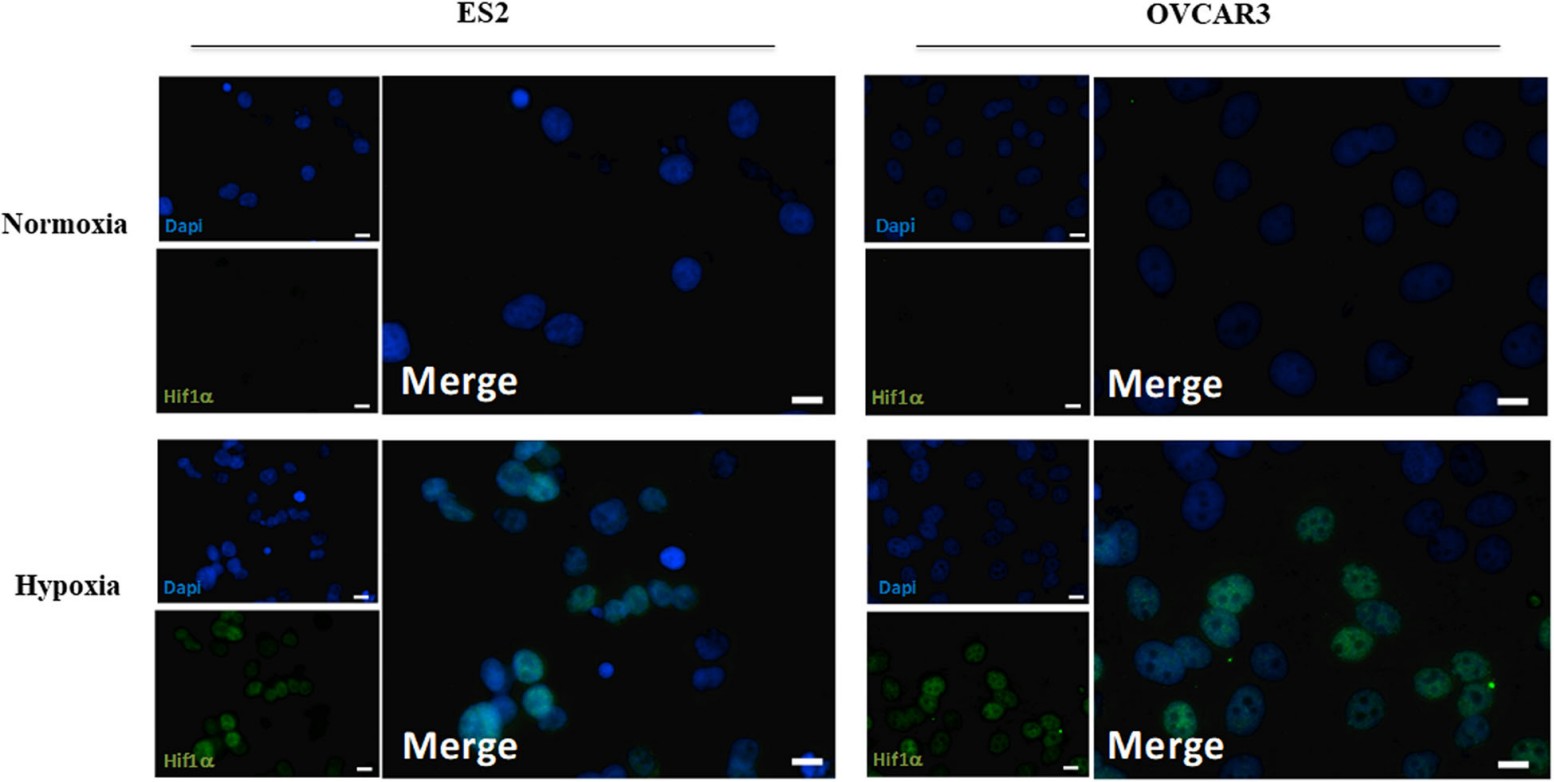
CoCl_2_ induces HIF-1α expression in ES2 cells and OVCAR3 cells. Immunofluorescence analysis of Hif-1α expression (green) under normoxia and hypoxia for ES2 and OVCAR3 cells. Nuclei were stained with DAPI (blue). White bars scale mean 20 μm

We started by confirming the induction of HIF1α expression by CoCl_2_. In fact, HIF1α expression was increased in both cell lines upon exposure to CoCl_2_ (Fig. 1).

Then, we have assessed the selective effects of normoxia (N) and CoCl_2_ mimicked hypoxia (H) in ES2 (OCCC) and OVCAR3 (OSC) cells proliferation, assessed by trypan blue staining and counting under a light microscope. The codes of each cell line and culture condition are presented in Table 1.

**Fig. 2:**
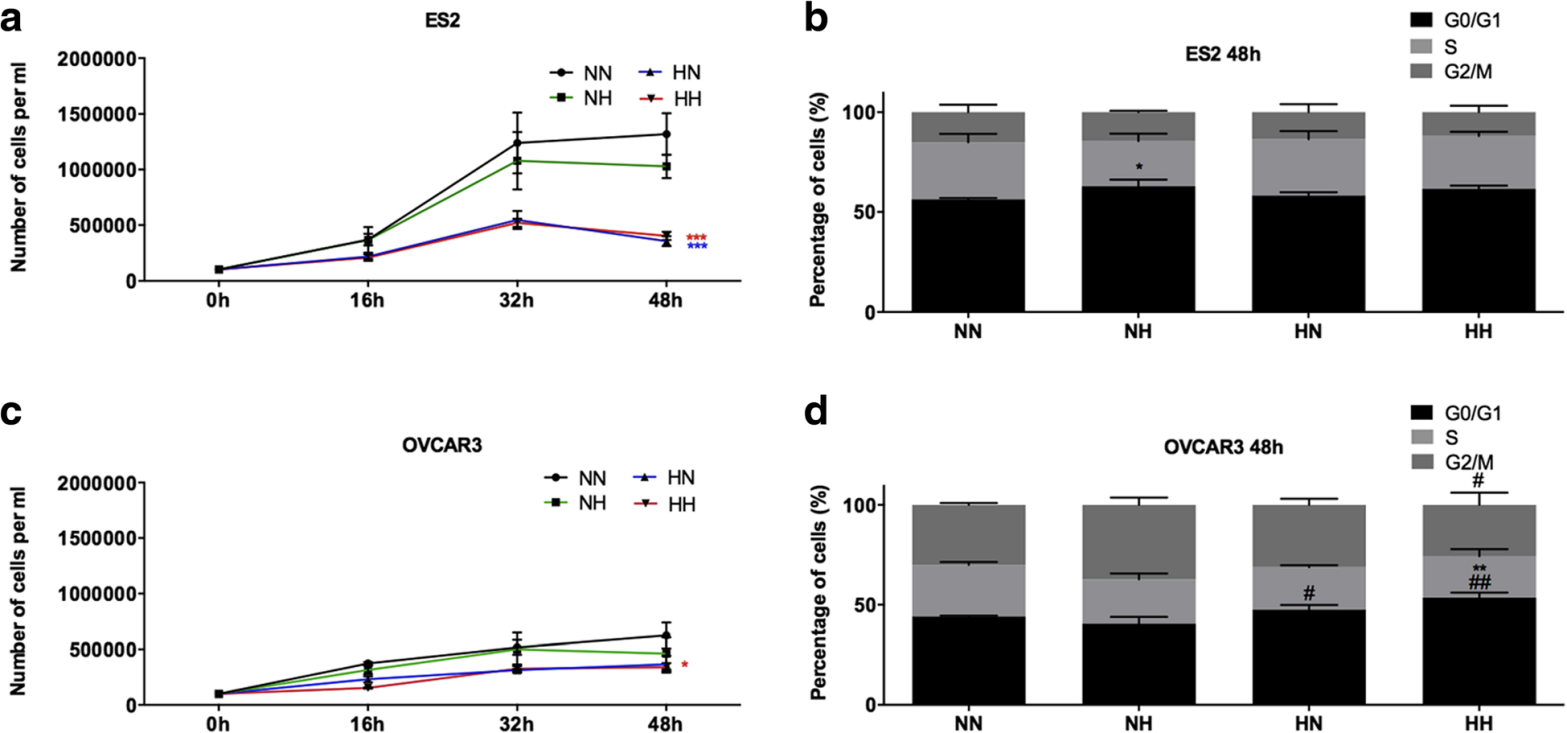
Proliferation rate of ES2 (OCCC) and OVCAR3 (OSC) cells selected under normoxia and under CoCl_2_ mimicked hypoxia. **a**. Proliferation curve for ES2 cells, **b**. Cell cycle analysis for ES2 cells for 48 h of assay, **c**. Proliferation curve for OVCAR3 cells, and **d**. Cell cycle analysis for OVCAR3 cells for 48 h of assay. NN – cells selected under normoxia and cultured under normoxia; NH – cells selected under normoxia and cultured under CoCl_2_ mimicked hypoxia; HN – cells selected under hypoxia and cultured under normoxia; HH – cells selected under CoCl_2_ mimicked hypoxia and cultured under CoCl_2_ mimicked hypoxia. Asterisks represent statistical significance in comparison with cells selected under normoxia and cultured under normoxia (NN). Cardinals represent statistical significance in comparison with cells selected under normoxia and cultured under hypoxia (NH). *p < 0.05, **p < 0.01, ***p < 0.001 (One-way ANOVA with post hoc Tukey tests)

The proliferation curves showed that ES2-N cells proliferated more than ES2-H, both in N and in H (Table 2 and Fig. 2a). In addition, ES2-NN tended to proliferate more than ES2-NH which is supported by cell cycle analysis, which showed that ES2-NN had a lower percentage of cells in G0/G1 than ES2-NH (Fig. 2b). Cell cycle analysis was performed by flow cytometry using PI staining in ethanol fixed cells.

**Table 2:** Adaptation to normoxia (N) confers a highly proliferative ability to ES2 cells

A.	
Treatments – proliferation curve (48 h)	Tukey test sig.
ES2-NN vs ES2-HN	0.000
ES2-NN vs ES2-HH	0.000
ES2-NH vs ES2-HN	0.000
ES2-NH vs ES2-HH	0.001
B.	
Treatments – cell cycle analysis	Tukey test sig.
G0/G1 ES2-NN vs ES2-NH	0.02
C.	
Treatments – proliferation curve (48 h)	Tukey test sig.
OVCAR3-NN vs OVCAR3-HH	0.036
D.	
Treatments – cell cycle analysis	Tukey test sig.
G0/G1 OVCAR3-NN vs OVCAR3-HH	0.006
G0/G1 OVCAR3-NH vs OVCAR3-HN	0.033
G0/G1 OVCAR3-NH vs OVCAR3-HH	0.001
G2/M OVCAR3-NH vs OVCAR3-HH	0.027

Regarding OVCAR3 cells, OVCAR3-NN proliferated more than OVCAR3-HH (Fig. 2c). The cell cycle analysis showed that OVCAR3-HH presented a higher percentage of cells in G0/G1 than both OVCAR3-NN and OVCAR3-NH (Fig. 2d).

### Adaptation to normoxia (N) is accompanied by an evolutionary trade-off that is suppressed by cysteine under CoCl_2_ mimicked hypoxia (H) in ES2 cells

Cell death, by flow cytometry using annexin V and popidium iodide (PI) staining, was used to assess the selective effects of N and H in ES2 (OCCC) and OVCAR3 (OSC). The codes of each cell line and culture condition are presented in Table 1.

**Fig. 3:**
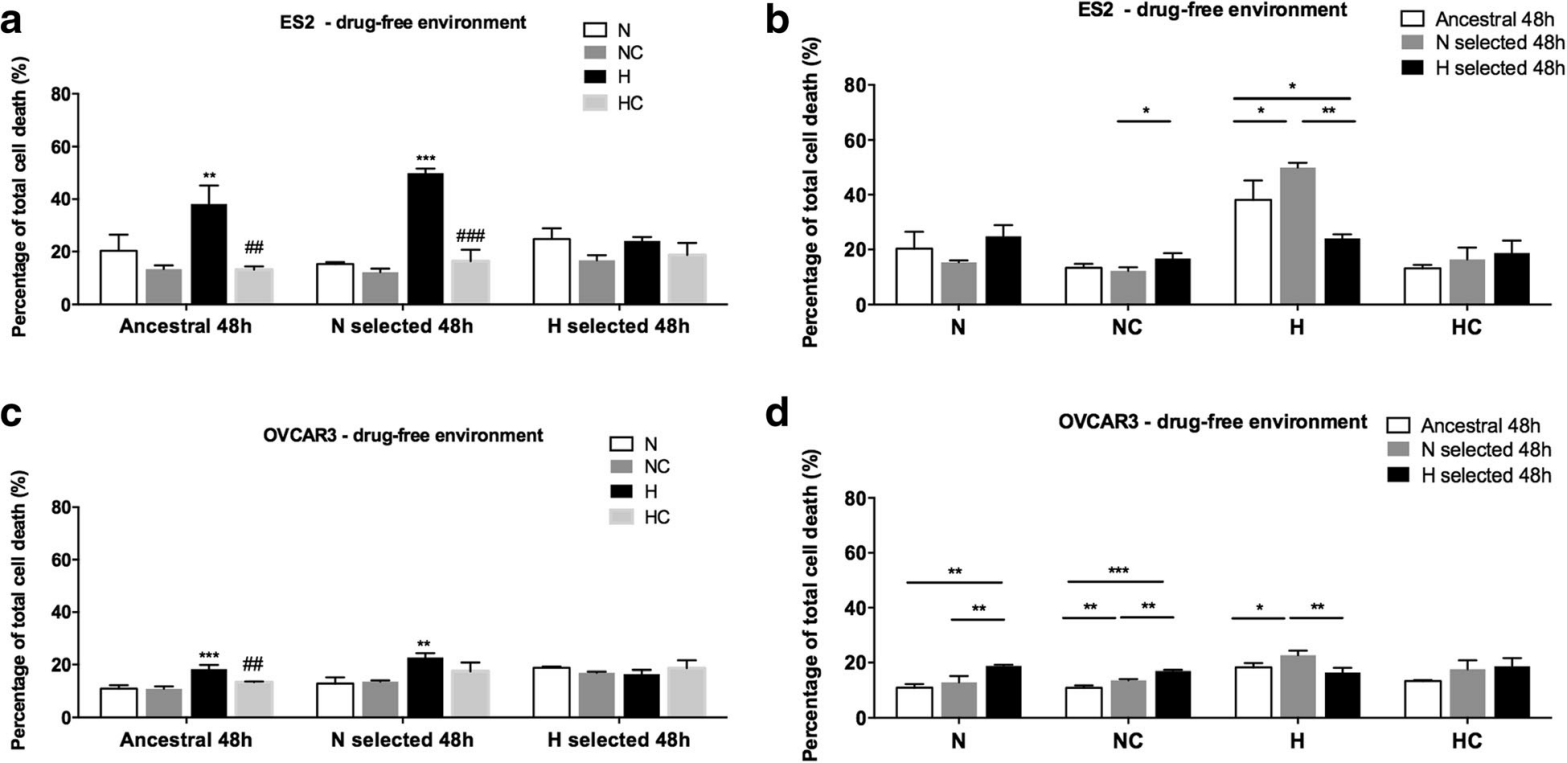
Adaptation to normoxia (N) is accompanied by an evolutionary trade-off which is supressed by cysteine under CoCl_2_ mimicked hypoxia (H) in ES2 cells. Cell death levels in a drug-free environment for **a**. and **b**. ES2 cells and **c**. and **d**. OVCAR3 cells. N selected – cells selected under normoxia; H selected – cells selected under CoCl_2_ mimicked hypoxia; N – Normoxia; NC – Normoxia supplemented with cysteine; H – CoCl_2_ mimicked hypoxia; HC – CoCl_2_ mimicked hypoxia supplemented with cysteine. Results are shown as mean ± SD. In **a**. and **c**. asterisks represent statistical significance compared to cells cultured under normoxia within each cell line. Cardinals (#) represent statistical significance of cells cultured in CoCl_2_ mimicked hypoxia with cysteine compared to cells cultured under CoCl_2_ mimicked hypoxia without cysteine within each cell line. In **b**. and **d**. asterisks represent statistical significance among cell lines within each treatment. *p < 0.05, **p < 0.01, ***p < 0.001 or #*p* < 0.05, ##*p* < 0.01, ###*p* < 0.001 (One-way ANOVA with post hoc Tukey tests)

Cell death analysis showed that ES2-A, ES2-N and ES2-H have a trend to benefit from cysteine in normoxia. However, ES2-AH and ES2-NH benefit from cysteine, having lower cell death levels. ES2-N showed to be more sensitive to CoCl_2_ mimicked hypoxia than ES2-A thus showing an evolutionary trade-off in the adaptation to N; and cysteine was able to supress this trade-off (Fig. 3a, b and Table 3A, B). As expected for ES2-H any Tukey test presented statistical significance amongst conditions, suggesting that ES2-H performed equally in all environments (Fig. 3a, b and Table 3A, B).

**Table 3:** Adaptation to normoxia (N) is accompanied by an evolutionary trade-off that is suppressed by cysteine under CoCl2 mimicked hypoxia (H) in ES2 cells

A.	
Treatments – cell death analysis (48 h)	Tukey test sig.
ES2-AN vs ES2-AH	0.008
ES2-ANC vs ES2-AH	0.001
ES2-AHC vs ES2-AH	0.001
ES2-NN vs ES2-NH	0.000
ES2-NNC vs ES2-NH	0.000
ES2-NHC vs ES2-NH	0.000
B.	
Treatments – cell death analysis (48 h)	Tukey test sig.
ES2-NNC vs ES2-HNC	0.030
ES2-NH vs ES2-AH	0.034
ES2-NH vs ES2-HH	0.001
ES2-HH vs ES2-AH	0.016
C.	
Treatments – cell death analysis (48 h)	Tukey test sig.
OVCAR3-AN vs OVCAR3-AH	0.000
OVCAR3-ANC vs OVCAR3-AH	0.000
OVCAR3-AHC vs OVCAR3-AH	0.003
OVCAR3-NH vs OVCAR3-NN	0.003
OVCAR3-NH vs OVCAR3-NNC	0.004
D.	
Treatments – cell death analysis (48 h)	Tukey test sig.
OVCAR3-AN vs OVCAR3-HN	0.002
OVCAR3-NN vs OVCAR3-HN	0.009
OVCAR3-ANC vs OVCAR3-NNC	0.004
OVCAR3-ANC vs OVCAR3-HNC	0.000
OVCAR3-NNC vs OVCAR3-HNC	0.001
OVCAR3-AH vs OVCAR3-NH	0.036
OVCAR3-HH vs OVCAR3-NH	0.008

In normoxia, OVCAR3-A, OVCAR3-N and OVCAR3-H showed no differences in the absence and presence of cysteine. OVCAR3-N also showed to be more sensitive to CoCl_2_ mimicked hypoxia than OVCAR3-A, thus showing again an evolutionary trade-off in the adaptation to normoxia (Fig. 3c, d and Table 3 C, D). Interestingly, only OVCAR3-A showed a benefit from cysteine in hypoxia, suggesting that selection under normoxia (OVCAR3-N) led to a decreased dependence on cysteine metabolism or to the loss of efficacy in taking advantage from cysteine.

OVCAR3-H was worse adapted to normoxia than OVCAR3-A and OVCAR3-N, but under H they performed better than OVCAR3-N (Fig. 3 c, d and Table 3 C, D), thus suggesting that this cell line also present a evolutionary trade-off in the adaptation to hypoxia mimicked with CoCl_2_ under normoxia. However, like ES2-H, in OVCAR3-H performed equally in all environments (Fig. 3c, d).

We must highlight that there was no difference in the response to hypoxia mimicked with CoCl_2_ among OVCAR3 and the respective ES2 cells. Nevertheless, OVCAR3 cells presented lower cell death levels in this treatment compared to the respective ES2 cells (Additional file 1: Figure S1A and B and Additional file 2: Table S1).

### Metabolic evolution driven by CoCl_2_ mimicked hypoxia (H) provides stronger resistance to carboplatin

In here, we assessed the effects of selection under N and H on cells capacity to survive upon carboplatin exposure. The codes of each cell line and culture condition are presented in Table 1 and cell death was assessed by flow cytometry using annexin V and popidium iodide (PI) staining.

**Fig. 4:**
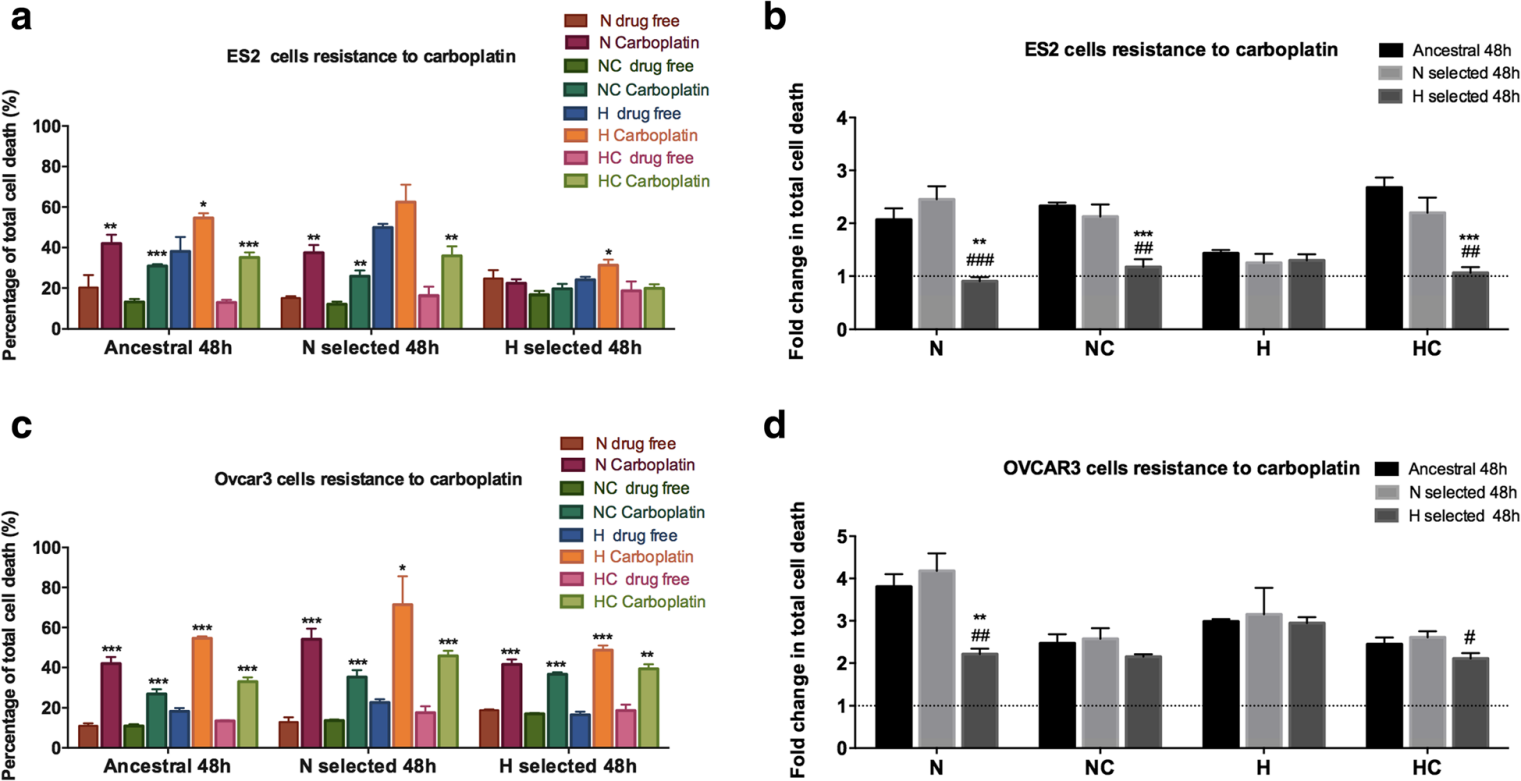
Metabolic evolution driven by hypoxia mimicked with CoCl_2_ provides stronger response to carboplatin cytotoxicity. Cells response to Carboplatin exposure for 48 h of assay for **a**. ES2 cells with non-normalized to control values, **b**. ES2 cells in which values were normalized to the respective control, **c**. OVCAR3 cells with non-normalized to control values and **d**. OVCAR3 cells in which values were normalized to the respective control. N selected – cells selected under normoxia; H selected – cells selected under hypoxia; N – Normoxia; NC – Normoxia supplemented with cysteine; H – Hypoxia mimicked with CoCl_2_; HC – Hypoxia mimicked with CoCl_2_ supplemented with cysteine. In **a**. and **c**. asterisks represent statistical significance compared to the respective control (cells cultured in the same experimental condition but in a free-drug environment) within each cell line. In **b**. and **d**. Asterisks represent statistical significance compared to ancestral cells. Cardinals (#) represent statistical significance compared to N-selected cells. Data were normalized to the respective control. Results are shown as mean ± SD. *p < 0.05, **p < 0.01, ***p < 0.001 or #*p* < 0.05, ##*p* < 0.01, ###*p* < 0.001 (A. and C. Independent samples T test and B. and D. One-way ANOVA with post hoc Tukey tests)

Upon carboplatin exposure, cell death levels increased for ES2-A cells in all treatments when compared to a drug-free environment. ES2-N cells showed a trend similar to ES2-A, in all conditions, with the exception of ES2-NH, in which there was a tendency for higher cell death levels upon carboplatin exposure, though not statistically significant (Fig. 4a and Table 4 A). Nonetheless, cysteine was advantageous under H in the presence of carboplatin for both ES2-A and ES2-N (Additional file 3: Figure S2 and Additional file 2: Table S2A). Interestingly, for ES2-H cells, upon carboplatin exposure, only ES2-HH showed a slight increase in cell death levels upon carboplatin (Fig. 4a and Table 4 A). Hence ES2-H cells present a higher survival capacity upon carboplatin exposure than ES2-A and ES2-N cells (Fig. 4b and Table 4 C). Interestingly, under H, no differences were observed among ES2-A, ES2-N and ES-H in carboplatin response, suggesting that 48 h of H exposure were sufficient to drive carboplatin response in ES2 cells, independent of the regime of selection.

**Table 4:** Hypoxia mimicked with CoCl_2_ provides stronger resistance to carboplatin

A.	
Treatments – cell death analysis (48 h)	Tukey test sig.
ES2-AN Ctr vs Carbopltin	0.008
ES2-ANC Ctr vs Carbopltin	0.000
ES2-AH Ctr vs Carbopltin	0.019
ES2-AHC Ctr vs Carbopltin	0.0000
ES2-NN Ctr vs Carbopltin	0.008
ES2-NNC Ctr vs Carbopltin	0.002
ES2-NH Ctr vs Carbopltin	0.069
ES2-NHC Ctr vs Carbopltin	0.006
ES2-HN Ctr vs Carbopltin	0.412
ES2-HNC Ctr vs Carbopltin	0.175
ES2-HH Ctr vs Carbopltin	0.016
ES2-HHC Ctr vs Carbopltin	0.706
B.	
Treatments – cell death analysis (48 h)	Tukey test sig.
ES2-HN vs ES2-AN	0.001
ES2-HN vs ES2-NN	0.000
ES2-HNC vs ES2-ANC	0.000
ES2-HNC vs ES2-NNC	0.001
ES2-HHC vs ES2-AHC	0.000
ES2-HHC vs ES2-NHC	0.001
C.	
Treatments – cell death analysis (48 h)	Tukey test sig.
OVCAR3-AN Ctr vs Carbopltin	0.000
OVCAR3-ANC Ctr vs Carbopltin	0.000
OVCAR3-AH Ctr vs Carbopltin	0.000
OVCAR3-AHC Ctr vs Carbopltin	0.000
OVCAR3-NN Ctr vs Carbopltin	0.000
OVCAR3-NNC Ctr vs Carbopltin	0.000
OVCAR3-NH Ctr vs Carbopltin	0.026
OVCAR3-NHC Ctr vs Carbopltin	0.000
OVCAR3-HN Ctr vs Carbopltin	0.000
OVCAR3-HNC Ctr vs Carbopltin	0.000
OVCAR3-HH Ctr vs Carbopltin	0.000
OVCAR3-HHC Ctr vs Carbopltin	0.001
D.	
Treatments – cell death analysis (48 h)	Tukey test sig.
OVCAR3-HN vs OVCAR3-AN	0.002
OVCAR3-HN vs OVCAR3-NN	0.001
OVCAR3-HHC vs OVCAR3-NHC	0.013

Strikingly, when compared to the other selection regimes, cysteine was advantageous both under normoxia and hypoxia for the ES2 long term adaptated to hypoxia mimicked with CoCl_2_ cells (ES2-H) (Fig. 4a, b and Table 4 A, C). Moreover, within the long term hypoxia mimicked with CoCl_2_ selected ES2-H cells no differences were observed between ES2-HH and ES2-HHC or ES2-HN and ES2-HNC, showing once again that ES2-H tend to performed equally in all environements (Additional file 3: Figure S2 and Additional file 2: Table S2). This fact is reinforced by the results of ES2 cells selected in normoxia (ES2-N) showing that these cells present higher ratio of cell death when cultured in hypoxia mimicked with CoCl_2_ with cysteine (ES2-NHC) versus without cysteine (ES2-NH), upon carboplatin (Fig. 4). Together, results suggest that cysteine facilitates the adaptation to hypoxia mimicked with CoCl_2_, which, in turn, drives carboplatin resistance. On the contrary, long term normoxia drives the selection of cells that have less capacity of benefiting from cysteine protection under hypoxia and upon drug exposure.

OVCAR3 cells presented higher cell death levels upon carboplatin exposure in OVCAR3-A, OVCAR3-N and OVCAR3-H cells, when compared to a drug-free environment and in all treatments (Fig. 4c, and Table 4 D). Nonetheless, cysteine was advantageous under H in the presence of carboplatin for all OVCAR3 cells (Additional file 3: Figure S2 and Additional file 2: Table S2 B). Interestingly, OVCAR3-HN cells presented stronger survival ability upon carboplatin than OVCAR3-AN and OVCAR3-NN (Fig. 4d and Table 4 F). Taken together, results suggest that H-selection can also be advantageous for OVCAR3 cells upon carboplatin exposure, nonetheless at a lessen extent than ES2 cells.

### Carboplatin resistance driven by CoCl_2_ mimicked hypoxia is stronger in ES2 (OCCC) cells

**Fig. 5:**
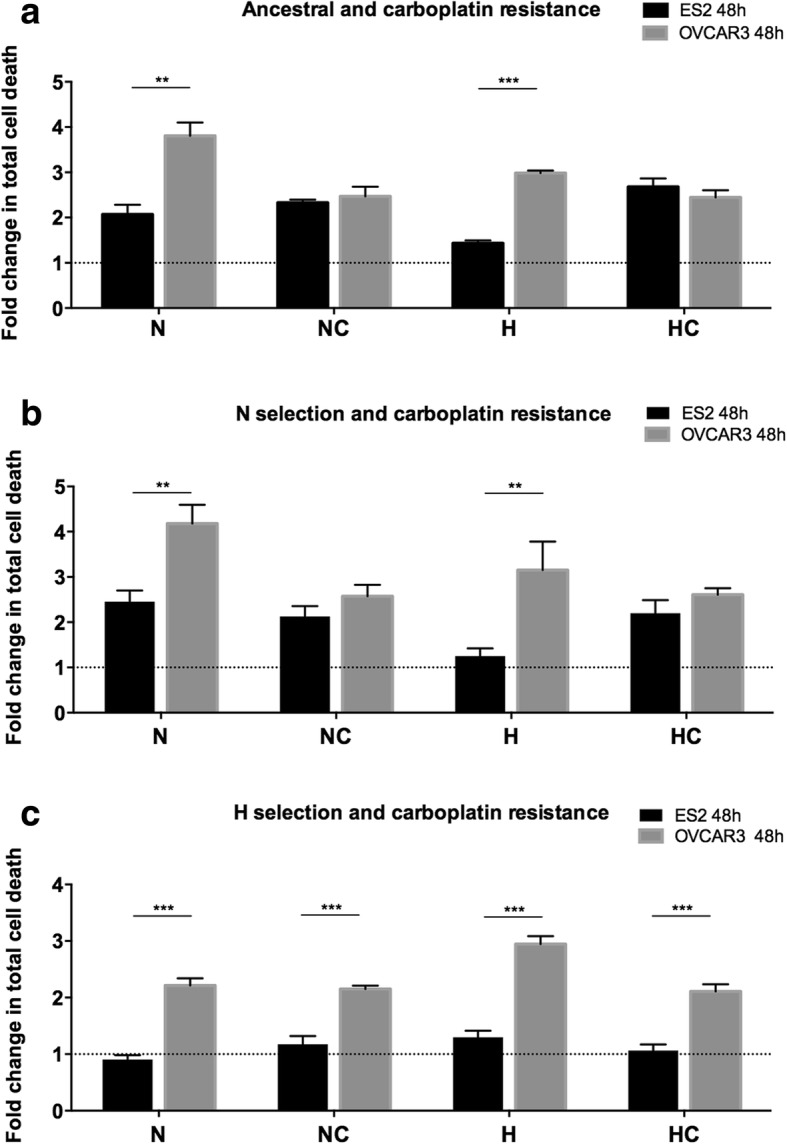
ES2 cells tend to present a stronger response to carboplatin than OVCAR3 cells. Comparison of ES2 amd OVCAR3 cells response to carboplatin for 48 h of experimental conditions for **a**. ancestral ES2 and OVCAR3 cells. **b**. ES2 and OVCAR3 cells selected under normoxia and **c**. ES2 and OVCAR3 cells selected under hypoxia mimicked with CoCl_2_. N – Normoxia; NC – Normoxia supplemented with cysteine; H – Hypoxia mimicked with CoCl_2_; HC – Hypoxia mimicked with CoCl_2_ supplemented with cysteine. Data were normalized to the respective control. Results are shown as mean ± SD. *p < 0.05, **p < 0.01, ***p < 0.001 (Independent samples T test)

We next compared ES2 and OVCAR3 ancestral and selected cells response dynamics to carboplatin exposure. Results of cell death analysis, by means of flow cytometry and annexin V and propidium iodide (PI) staining, showed that ES2-A cells presented a stronger resistance to carboplatin both under N and H than OVCAR3-A cells for 48 h of assay (Fig. 5a and Table 5 A). Similar results were observed for N selected cell lines, where ES2-NN and ES2-NH presented a stronger carboplatin resistance compared to OVCAR3-NN and OVCAR3-NH cells (Fig. 5b and Table 5 B). Interestingly, ES2-H cells presented a stronger resistance to carboplatin in all treatments when compared to OVCAR3-H cells (Fig. 5c and Table 5 C).

**Table 5:** ES2 cells tend to present a stronger resistance to carboplatin than OVCAR3 cells

A.	
Treatments – cell death analysis (48 h)	T test sig.
ES2-AN vs OVCAR3-AN	0.001
ES2-ANC vs OVCAR3-ANC	0.347
ES2-AH vs E OVCAR3-AH	0.000
ES2-AHC vs OVCAR3-AHC	0.179
B.	
Treatments – cell death analysis (48 h)	T test sig.
ES2-NN vs OVCAR3-NN	0.003
ES2-NNC vs OVCAR3-NNC	0.085
ES2-NH vs E OVCAR3-NH	0.007
ES2-NHC vs OVCAR3-NHC	0.092
C.	
Treatments – cell death analysis (48 h)	T test sig.
ES2-HN vs OVCAR3-HN	0.000
ES2-HNC vs OVCAR3-HNC	0.000
ES2-HH vs E OVCAR3-HH	0.000
ES2-HHC vs OVCAR3-HHC	0.000

**Fig. 6:**
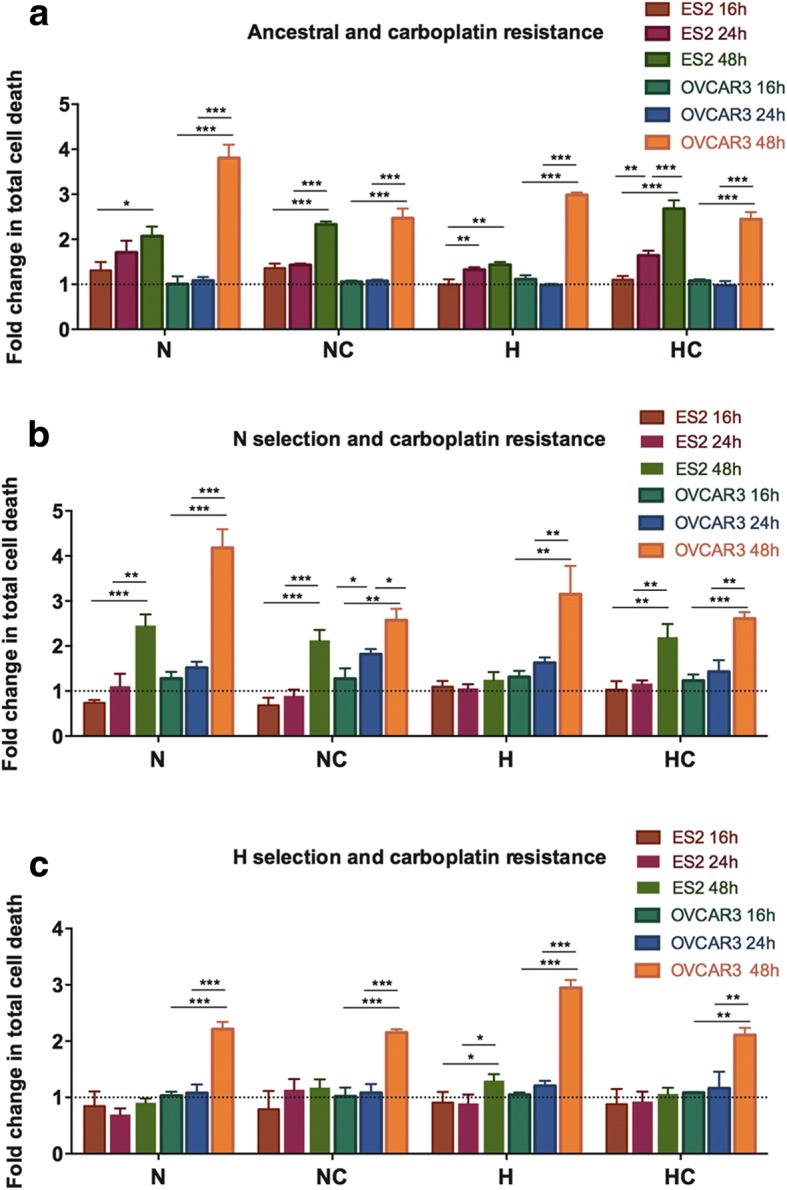
ES2 (OCCC) and OVCAR3 (OSC) cells present different dynamics of response to carboplatin. Cells response to Carboplatin over time for **a**. ancestral ES2 cells and OVCAR3 cells, **b**. ES2 and OVCAR3 cells selected under normoxia, **c**. ES2 and OVCAR3 cells selected under hypoxia mimicked with CoCl_2_. N – Normoxia; NC – Normoxia supplemented with cysteine; H – Hypoxia mimicked with CoCl_2_; HC – Hypoxia mimicked with CoCl_2_ supplemented with cysteine. Data were normalized to the respective control. Results are shown as mean ± SD. *p < 0.05, **p < 0.01, ***p < 0.001 (One-way ANOVA with post hoc Tukey tests)

In ancestral cells, the dynamics of carboplatin response were similar between ES2 and OVCAR3 cells, in which carboplatin induced cell death in a time-dependet manner (Fig. 6a and Table 6 A). However, ES2-NH cells showed a stable response to carboplatin over time, whereas OVCAR3-NH cells also presented increased cell death levels with increasing time of carboplatin exposure (Fig. 6b and Table 6 B). In all conditions, ES2-H cells showed a stable carboplatin response, with the exception of ES2-HH, in which carboplatin induced a slight increase in cell death levels with increasing time of exposure. In OVCAR3-H cells, carboplatin induced cell death in a time-dependet manner in all treatments (Fig. 6c and Table 6 C).

**Table 6:** ES2 and OVCAR3 cells dynamics of response to carboplatin

A.	
Treatments – cell death analysis - 16 h vs 24 h vs 48 h	Tukey test sig.
ES2-AN 16 h vs 48 h	0.014
ES2-ANC 16 h vs 48 h	0.000
ES2-ANC 24 h vs 48 h	0.000
ES2-AH 16 h vs 24 h	0.006
ES2-AH 24 h vs 48 h	0.001
ES2-AHC 16 h vs 24 h	0.007
ES2-AHC 16 h vs 48 h	0.000
ES2-AHC 24 h vs 48 h	0.000
OVCAR3-AN 16 h vs 48 h	0.000
OVCAR3-AN 24 h vs 48 h	0.000
OVCAR3-ANC 16 h vs 48 h	0.000
OVCAR3-ANC 24 h vs 48 h	0.000
OVCAR3-AH 16 h vs 48 h	0.000
OVCAR3-AH 24 h vs 48 h	0.000
OVCAR3-AHC 16 h vs 48 h	0.000
OVCAR3-AHC 24 h vs 48 h	0.000
B.	
Treatments – cell death analysis - 16 h vs 24 h vs 48 h	Tukey test sig.
ES2-NN 16 h vs 48 h	0.000
ES2-NN 24 h vs 48 h	0.001
ES2-NNC 16 h vs 48 h	0.000
ES2-NNC 24 h vs 48 h	0.000
ES2-NHC 16 h vs 48 h	0.001
ES2-NHC 24 h vs 48 h	0.002
OVCAR3-NN 16 h vs 48 h	0.000
OVCAR3-NN 24 h vs 48 h	0.000
OVCAR3-NNC 16 h vs 24 h	0.041
OVCAR3-NNC 16 h vs 48 h	0.001
OVCAR3-NNC 24 h vs 48 h	0.01
OVCAR3-NH 16 h vs 48 h	0.002
OVCAR3-NH 24 h vs 48 h	0.006
OVCAR3-NHC 16 h vs 48 h	0.000
OVCAR3-NHC 24 h vs 48 h	0.001
C.	
Treatments – cell death analysis - 16 h vs 24 h vs 48 h	Tukey test sig.
ES2-HH 16 h vs 48 h	0.048
ES2-HH 24 h vs 48 h	0.043
OVCAR3-HN 16 h vs 48 h	0.000
OVCAR3-HN 24 h vs 48 h	0.000
OVCAR3-HNC 16 h vs 48 h	0.000
OVCAR3-HNC 24 h vs 48 h	0.000
OVCAR3-HH 16 h vs 48 h	0.000
OVCAR3-HH 24 h vs 48 h	0.000
OVCAR3-HHC 16 h vs 48 h	0.001
OVCAR3-HHC 24 h vs 48 h	0.002

Taken together, results suggest that hypoxia mimicked with CoCl_2_ (H) drives carboplatin resistance in ES2 and, at a lower extent, in OVCAR3 cells, thus pointing a more aggressive phenotype in ES2-H than in OVCAR3-H cells. Since ES2-A cells and ES2-N cells were able to take advantage from cysteine in H (ES2-AH and ES2-NH), we propose that cysteine allows a quicker response and adaptation to H conditions that, in turn, drive carboplatin resistance.

### ES2 cells present metabolic diversity in adverse environments, favouring resistance to carboplatin

The codes of each cell line and culture condition are presented in Table 1.

**Fig. 7:**
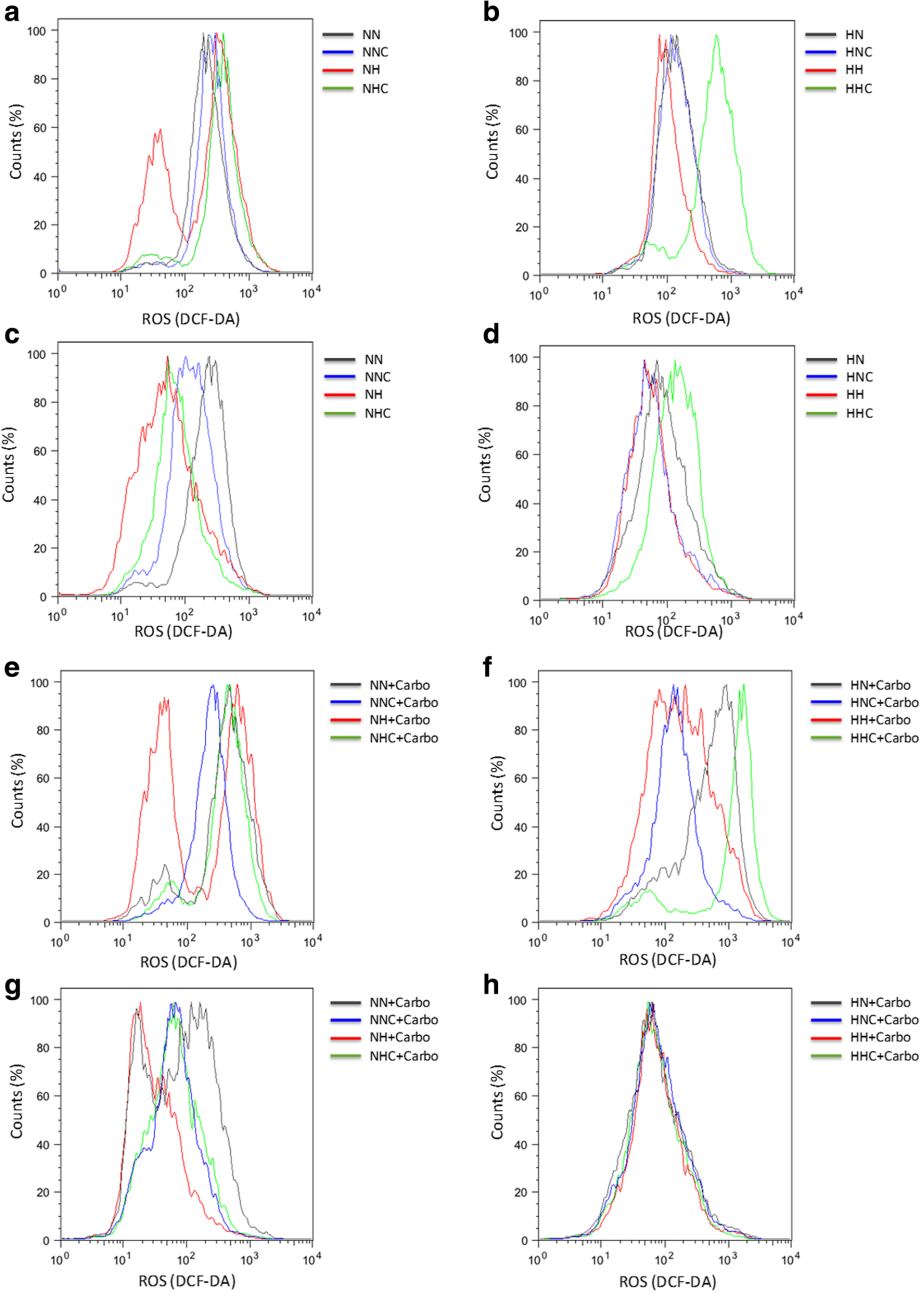
ES2 cells present metabolic diversity in adverse environments. ROS histogram for **a**. ES2-N cells, **b**. ES2-H cells, **c**. OVCAR3-N cells, **d**. OVCAR3-H cells in a free drug environment for 48 h of experimental conditions, and ROS histogram for **e**. ES2-N cells, **f**. ES2-H cells, **g**. OVCAR3-N cells and **h**. OVCAR3-H in the presence of carboplatin. NN – cells selected under normoxia and cultured under normoxia (grey line); NNC – cells selected under normoxia and cultured under normoxia supplemented with cysteine (blue line); NH – cells selected under normoxia and cultured under hypoxia mimicked with CoCl_2_ (red line); NHC – cells selected under normoxia and cultured under hypoxia mimicked with CoCl_2_ supplemented with cysteine (green line); HN – cells selected under hypoxia mimicked with CoCl_2_ and cultured under normoxia (grey line); HNC – cells selected under hypoxia mimicked with CoCl_2_ and cultured under normoxia supplemented with cysteine (blue line); HH – cells selected under hypoxia mimicked with CoCl_2_ and cultured under hypoxia (red line); HHC – cells selected under hypoxia and cultured under hypoxia mimicked with CoCl_2_ supplemented with cysteine (green line)

In a drug-free environment, the analysis of ROS levels by flow cytometry analysis allowed the observation of two distinct populations in ES2-NH (Fig. 7a), suggesting the existence of a glycolytic and an oxidative phosphorylative population of cells. Interestingly, hypoxia mimicked with CoCl_2_ was especially disadvantageous for those cells, presenting the higher cell death levels in this condition (Fig. 3a). This suggests that metabolic diversity among ES2-N cells could be a strategy to cope with new adverse environments.

In ES2-HH, we only observed one population, thus revelling a higher metabolic adaptive capacity to H (Fig. 7b). Interestingly, in OVCAR3-NH we were not able to distinguish two different populations of cells as in ES2-NH (Fig. 7c). Also, we observed a trend to higher ROS levels in both ES2-N and ES2-H than in OVCAR3-N and OVCAR3-H, especially in conditions with cysteine supplementation (Additional file 4: Figure S3A to D and Additional file 2: Table S3 A to D). This might indicate that cysteine allows higher metabolic activity in ES2 cells, even under H. Moreover, the detection of ROS, using 2′, 7′-Dichlorofluorescin diacetate, never showed a correlation between higher ROS levels and higher cell death levels in any cell line. On the contrary, ROS showed a negative correlation with cell death.

Upon carboplatin exposure, different populations were also observed for ES2-N cells under H (Fig. 7e), thus showing again that this cell line present different cell populations with different metabolic states in an adverse environment. In addition, ES2-HH with cysteine showed a notable increase in ROS levels upon carboplatin exposure (Fig. 7f, Additional file 4: Figure S3E and F and Additional file 2: Table S3 E and F). Upon carboplatin exposure, OVCAR3-A, OVCAR3-N and OVCAR3-H cells did not show different populations in any treatment (Fig. 7g and h). Interestingly, OVCAR3-H selected cells showed no differences in ROS dynamics, thus suggesting that cells do not present metabolic diversity (Fig. 7h).

Taken together, results suggest that ES2 cells present higher metabolic diverse strategies under adverse environments when compared to OVCAR3 cells. This diversity possibly explains the increased response capacity to the more stressful environments (hypoxia mimicked with CoCl_2_ and carboplatin) of ES2 cells, whereas, in general, OVCAR3 cells failed to respond to it.

## Discussion

Although the outcome prognosis of OCCC and OSC had been a matter of controversy, it was shown that patients with OCCC had a significantly worse prognosis than patients with OSC when matched for age, stage, and level of primary surgical cytoreduction [36, 37]. Moreover, while OCCC shows primary resistance to conventional platinum-based chemotherapy, OSC at first shows sensitiveness [10, 11] with the development of progressive resistance [9]. In here, we used two different cancer cell lines derived from these two histological types of ovarian cancer and addressed the effect of cells selection under normoxia and CoCl_2_ mimicked hypoxia on the evolutionary outcome of cancer cells, exploring also the role of cysteine in this adaptive process.

It is widely accepted that adaptation to a specific environment is associated to deterioration in other non-selective environments, being accompanied by an evolutionary trade-off [38–41]. In fact, our results suggest that there is an evolutionary trade-off in ovarian cancer cells adaptation to normoxia conditions in which, cells adapted under normoxia duplicated rapidly but at the cost of increased mortality in adverse environments. Notably, in ES2 (OCCC) cells, cysteine was able to suppress this trade-off under CoCl_2_ mimicked hypoxia (ES2-NH versus ES2-NHC). Our previous data have shown that cysteine is able to protect cells from death under CoCl_2_ mimicked hypoxia, allowing fast adaptation to those conditions, especially in ES2 cells (unpublished data). Evidence suggests that intracellular cysteine directly induces the HIF prolyl-hydroxylases, leading to HIF-1α degradation [42, 43]. This suggests that cysteine is able to convert a hypoxic cellular metabolism into a normoxic one. In addition, our data suggests that ES2 ancestral cells present both higher intracellular cysteine and GSH degradation levels under hypoxia mimicked with CoCl_2_ supplemented with cysteine compared to hypoxia mimicked with CoCl_2_ without cysteine supplementation (data not shown). Those observations could explain the protective effect of cysteine under hypoxia in both ancestral and normoxia selected ES2 cells, thus allowing counteracting the trade-off under hypoxia. Those results also suggest that ES2 cells selected under normoxia (ES2-N) still present metabolic diversity concerning cysteine metabolism under hypoxic conditions (ES2-NH). Interestingly, OVCAR3-N cells showed less plasticity. Moreover, ES2-H presented increased survival in non-selective environments compared to cells selected under normoxia (ES2-N), suggesting a more aggressive phenotype in these cells, as they seem to exhibit a generalist phenotype, hence more adaptive. Remarkably, results showed that the increased survival was accompanied by lower proliferation rates. Life history theory proposes that cancer cells may be subjected to trade-offs between maximizing cell survival and cell growth, and that both strategies can be successful depending on the environmental conditions [44]. We observed that ES2–H proliferated more slowly than ES2-N, but, nevertheless, presented increased survival in the presence of carboplatin, a cytotoxic agent used in ovarian cancer conventional chemotherapy, thus showing again that life-history trade-offs may have clinical implications for cancer patients. Those results are in accordance with the observations that hypoxia promote tumour progression and resistance to therapy (reviewed by Vaupel and Mayer) [45], having a complex role in the hallmarks of human cancers [13, 46, 47]. Importantly and surprisingly, hypoxia is known to induce mitochondrial ROS levels [48, 49]. ROS levels are widely associated with tumour initiation, progression and chemoresistance [48, 50, 51]. Our results showed increased ROS levels in ES2-H cells under hypoxia mimicked with CoCl_2_ and cysteine supplementation upon carboplatin exposure. Interestingly, in the same conditions, ES2 cells showed a higher ability to survive upon carboplatin exposure. Nevertheless, it remains unclear if the increased ROS levels are responsible for carboplatin resistance or, on the contrary, if the higher cells adaptability to this environment leads cells to increased metabolic activity, thus increasing ROS levels.

Notably, OVCAR3-A and OVCAR-N cells showed to be less sensitive to hypoxia mimicked with CoCl_2_ than ES2-A and ES2-N cells. This observation would suggest that those cells are more prone to chemoresistance than ES2 cells. However, OVCAR3 cells presented a poorer response capacity to carboplatin, thus suggesting that resistance to hypoxia alone cannot explain the more aggressive phenotypes. OVCAR3 cells also presented decreased cells diversity concerning ROS levels in adverse environments. Our results highlight the role of hypoxia-induced chemoresistance in combination with metabolic diversity in cancer cells coping with adverse conditions. Whereas ES2 cells showed metabolic diversity, thus suggesting metabolism reprogramming in adverse conditions, OVCAR3 cells seemed to be inefficient in this process, thus preventing an increased survival upon carboplatin cytotoxicity.

We have to highlight that ES2 and OVCAR3 cells were selected during different times, due to a lower proliferation rate of OVCAR3 cells in CoCl_2_ mimicked hypoxia than ES2, which could explain, in part, the lower diversity observed in OVCAR3 selected cells, as these cells were selected during more time than ES2 cells. However, in what concerns carboplatin resistance, we would expect an association between higher selection time and higher levels of resistance. However, in a general way, OVCAR3 selected cells showed to be less resistant than ES2 selected cells. Moreover, our main propose was to compare the effect of selection under normoxia and CoCl_2_ mimicked hypoxia and cysteine supplementation in the dynamics of adaptation to carboplatin within each cell line (ES2/OVCAR3) and the time of selection was the same in these situations. Also, the ancestral OVCAR3 (OVCAR3-A) cells showed similar dynamics of response to carboplatin as selected cells, corroborating the results. The proliferation curves/cell cycle analysis and cell death analysis /ROS quantification were also performed with different selection times within each cell line but we did not aim to compare proliferation with cell death. The only speculation done was regarding ES2 cells selected under CoCl_2_ mimicked hypoxia and increased survival accompanied by lower proliferation rates. However, since proliferation curves were performed with increased selection time, it would be expected that the same selection time as cell death analysis, would lead to a more pronounced effect on decreased cell proliferation, given less time for adaptation.

Our second hypothesis that selection under CoCl2 mimicked hypoxia in ES2 (ES2-H) cells would favour a stronger ability of cells to benefit from cysteine under CoCl2 mimicked hypoxia showed to be false in a drug-free environment. Strikingly, in the presence of carboplatin, cysteine was especially advantageous to ES2-H, thus suggesting that they evolved mechanisms to a better usage of this amino acid in new adverse environments. In this study, we only focused on the role of cysteine supplementation in response to hypoxia and further response to carboplatin cytotoxicity. We did not adressed other amino acids since we were interested in cysteine as a sulphur source in hypoxia mimicked with CoCl_2_ and carboplatin resistance. However, in another study we showed that glutamine also played a role in GSH synthesis, as glutamine is a source of glutamate and glycine [25], supporting again the role of thiols in chemoresistance.

**Fig. 8:**
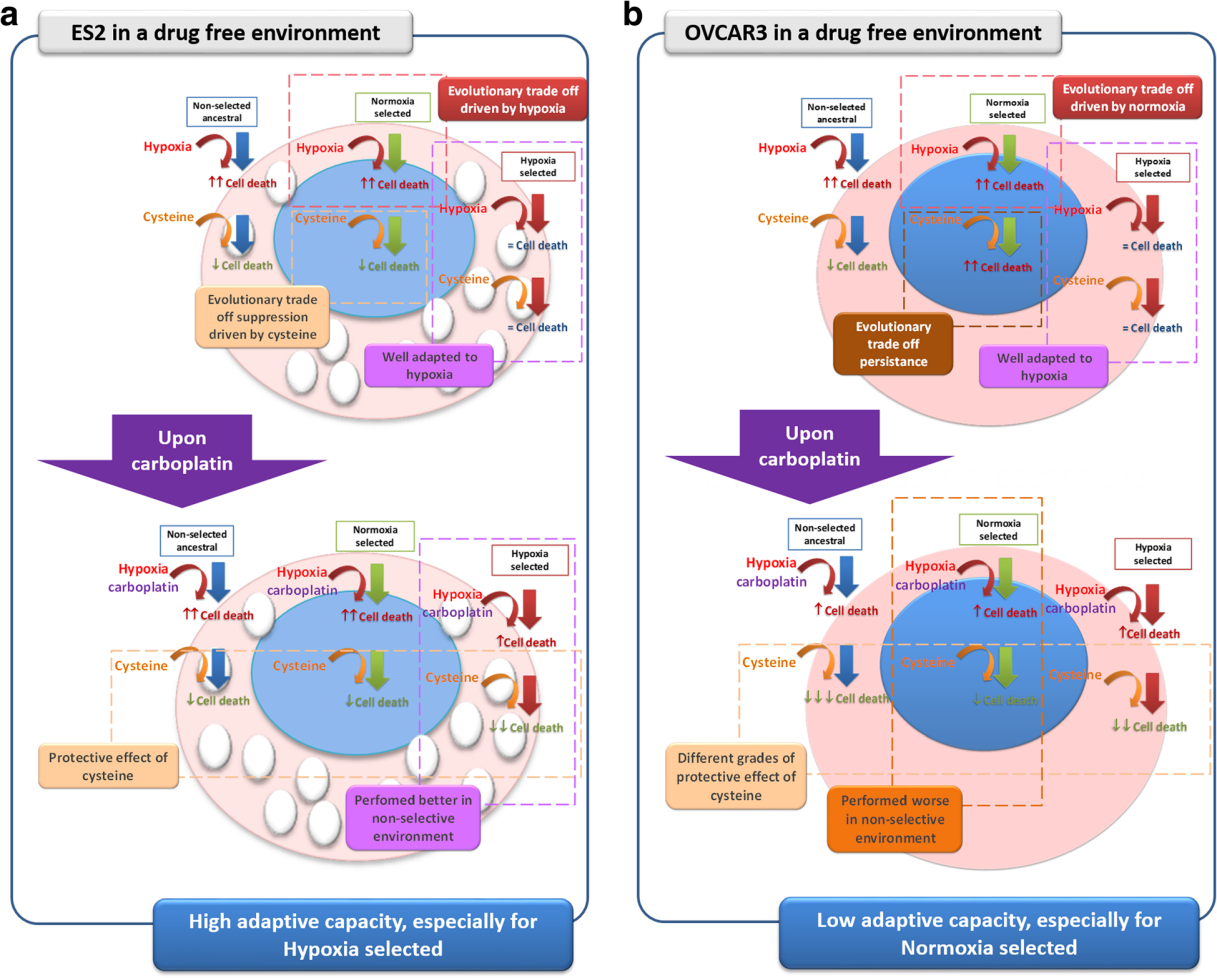
ES2 (OCCC) and OVCAR3 (OSC) showed different adaptive capacities in a drug free environment which influences the response to carboplatin. **a** Non-selected ancestral and Normoxia selected ES2 cell lines showed an evolutionary trade-off when exposed to CoCl_2_ mimicked hypoxia, this situation is reverted by the presence of cysteine. CoCl_2_ mimicked hypoxia selected cells behave equally in the presence or absence of cysteine. Upon carboplatin exposure, all ES2 cells variants benefits from a protective effect of cysteine, decreasing the cytotoxicity of carboplatin. So, ES2 cells exhibited a higher adaptive capacity to CoCl_2_ mimicked hypoxia and cysteine which reflects in a better fitness to the carboplatin rich non-selective environment, being CoCl_2_ mimicked hypoxia selected the best fitted. **b** Non-selected ancestral and Normoxia selected OVCAR3 cell lines showed an evolutionary trade-off when exposed to CoCl_2_ mimicked hypoxia, cysteine only reverts this situation in Non-selected ancestral cells. Upon carboplatin exposure, all OVCAR3 cells variants benefit from a protective effect of cysteine, decreasing the cytotoxicity of carboplatin. OVCAR3 cells variants benefit from different grades of cysteine protection: Non-selected ancestral>CoCl_2_ mimicked hypoxia selected>Normoxia selected. So, OVCAR3 cells exhibited a lower adaptive capacity to CoCl_2_ mimicked hypoxia and cysteine which reflects in a worse fitness to the carboplatin rich non-selective environment, being Normoxia selected the worst fitted. Overall, ES2 cells have a higher metabolic plasticity than OVCAR3 cells. This fact can underlie the intrinsic resistance to carboplatin exhibited by ES2 cancer in clinics. White ellipses in ES2 cells represent vacuoles characteristic of clear cell carcinoma

Taken together, results show that the adaptation to normoxia and CoCl_2_ mimicked hypoxia leads cancer cells to display opposite strategies. Whereas cells adapted to CoCl_2_ mimicked hypoxia tend to proliferate less but present increased survival in adverse environments, cells adapted to normoxia present the opposite strategy, proliferating rapidly but at the cost of increased mortality in adverse environments. Albeit the number of cell lines might be a limitation, we believe those different evolutionary courses might be in the future taken into account in the clinical context, as therapy protocols could be more effective dependent on the evolutionary strategy of cancer cells. Moreover, results stressed that the ability of cancer cells to use cysteine has an impact in cancer cells adaptation to a CoCl_2_ mimicked hypoxic environment and, ultimately, to platinum-based chemotherapeutic agents, allowing the selection of resistant phenotypes that are more aggressive, being able to carry out cancer progression and recurrence (Fig. 8).

Finally, our study pave the path to show that experimental evolution in cancer can be a valuable tool to predict the metabolic courses underlying resistance to drugs, which will contribute to an endpoint improvement of fighting cancer strategies.

## Conclusions

Despite the limitation of cell line models our results light the role of metabolic evolution driven by CoCl_2_ mimicked hypoxia selection and cysteine availability in ovarian cancer cells response to chemotherapy. Moreover, our results highlight cysteine bioavailability as a source of new therapeutic targets in order to reverse resistance both to hypoxia and carboplatin. Finally, the ability of cancer cells to metabolize and import cysteine could also be used to predict the development of resistance to platinum based therapy. Currently, we are developing a study to disclose the biochemical mechanism underlying the benefits of cysteine in order to find prognostic markers and ideally targets to overcome chemoresistance in ovarian cancer.

## Additional files

Additional file 1: **Figure S1.** ES2 and OVCAR3 cells resistance to hypoxia mimicked with CoCl_2_. Comparison of ES2 and OVCAR3 cells resistance to hypoxia mimicked with CoCl_2_ for 48 h of assay for A. ES2 and OVCAR3 cells in which values were normalized to the respective control, and B. ES2 and OVCAR3 cells with non-normalized to control values. N selected – cells selected under normoxia; H selected – cells selected under hypoxia mimicked with CoCl_2_. Results are shown as mean ± SD. Asterisks represent statistical significance between ES2 and OVCAR3 cells. *p < 0.05, **p < 0.01, ***p < 0.001 (Independent samples T tests). (TIFF 127 kb)

Additional file 2: **Table S1.** ES2 and OVCAR3 cells resistance to hypoxia mimicked with CoCl_2_. **Table S2.** Metabolic evolution driven by hypoxia mimicked with CoCl_2_ provides stronger resistance to carboplatin. **Table S3.** ROS levels in ES2 (OCCC) and OVCAR3 (OSC) ancestral cells, cells selected under normoxia and under hypoxia mimicked with CoCl_2_. (DOCX 16 kb)

Additional file 3: **Figure S2.** Metabolic evolution driven by hypoxia mimicked with CoCl_2_ provides stronger resistance to carboplatin. Cell death levels (non-normalized values) in the presence of carboplatin for 48 h of assay for A. ES2 cells and B. OVCAR3 cells. N selected – cells selected under normoxia; H selected – cells selected under hypoxia mimicked with CoCl_2_; N – Normoxia; NC – Normoxia supplemented with cysteine; H – Hypoxia mimicked with CoCl_2_; HC – Hypoxia mimicked with CoCl_2_ supplemented with cysteine. Results are shown as mean ± SD. Asterisks represent statistical significance compared to cells cultured under normoxia within each cell line. Cardinals represent statistical significance compared to cells cultured under hypoxia within each cell line. *p < 0.05, **p < 0.01, ***p < 0.001 or #p < 0.05, ##p < 0.01, ###p < 0.001 (One-way ANOVA with post hoc Tukey tests). (TIFF 159 kb)

Additional file 4: **Figure S3.** ROS levels in ES2 (OCCC) and OVCAR3 (OSC) ancestral cells, cells selected under normoxia and under hypoxia mimicked with CoCl_2_. ROS levels in a drug-free environment for 48 h of assay for A. and B. ES2 cells and C. and D. OVCAR3 cells and ROS levels in the presence of Carboplatin for 48 h of assay for E. and F. ES2 cells and G. and H. OVCAR3. N selected – cells selected under normoxia; H selected – cells selected under hypoxia mimicked with CoCl_2_; N – Normoxia; NC – Normoxia supplemented with cysteine; H – Hypoxia mimicked with CoCl_2_; HC – Hypoxia mimicked with CoCl_2_ supplemented with cysteine. Results are shown as mean ± SD. Asterisks represent statistical significance compared to cells cultured under normoxia within each cell line. Cardinals represent statistical significance compared to cells cultured under hypoxia mimicked with CoCl_2_ within each cell line. *p < 0.05, **p < 0.01, ***p < 0.001 or #p < 0.05, ##p < 0.01, ###p < 0.001 (One-way ANOVA with post hoc Tukey tests). (TIFF 484 kb)

## Abbreviations

A: Ancestral
H: CoCl_2_ mimicked hypoxia
HC: CoCl_2_ mimicked hypoxia with cysteine supplementation
HH: Selected in hypoxia and cultured in CoCl_2_ mimicked hypoxia
HHC: Selected in hypoxia and cultured in CoCl_2_ mimicked hypoxia with cysteine supplementation
HIF-1α: Hypoxia inducible factor 1α
HN: Selected in hypoxia and cultured in normoxia
HNC: Selected in hypoxia and cultured in normoxia with cysteine supplementation
N: Normoxia
NC: Normoxia with cysteine supplementation
NH: Selected in normoxia and cultured in CoCl_2_ mimicked hypoxia
NHC: Selected in normoxia and cultured in CoCl_2_ mimicked hypoxia with cysteine supplementation
NN: Selected in normoxia and cultured in normoxia
NNC: Selected in normoxia and cultured in normoxia with cysteine supplementation
OCCC: Ovarian clear cell carcinoma
OSC: Ovarian serous carcinoma
