# Supplementary figures and images for: Cysteine boosters the evolutionary adaptation to CoCl2 mimicked hypoxia conditions, favouring carboplatin resistance in ovarian cancer
Source: BMC Evol Biol. 2018 Jun 19;18:97. doi: 10.1186/s12862-018-1214-1 (PMC6011206; doi:10.1186/s12862-018-1214-1)

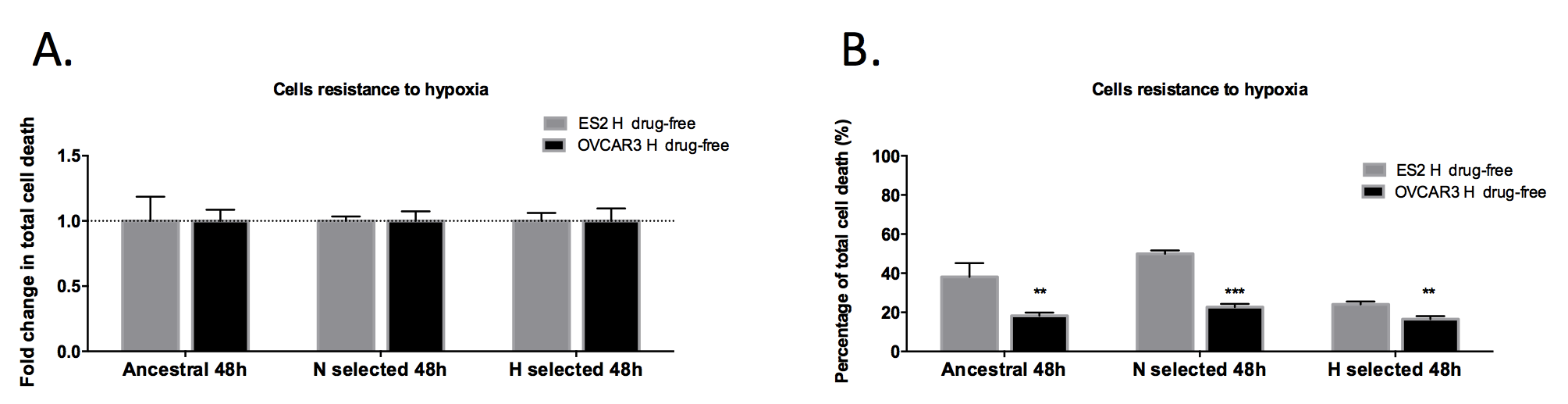

Supplement: Supplementary file 1 — Figure S1. ES2 and OVCAR3 cells resistance to hypoxia mimicked with CoCl2. Comparison of ES2 and OVCAR3 cells resistance to hypoxia mimicked with CoCl2 for 48 h of assay for A. ES2 and OVCAR3 cells in which values were normalized to the respective control, and B. ES2 and OVCAR3 cells with non-normalized to control values. N selected – cells selected under normoxia; H selected – cells selected under hypoxia mimicked with CoCl2. Results are shown as mean ± SD. Asterisks represent statistical significance between ES2 and OVCAR3 cells. *p < 0.05, **p < 0.01, ***p < 0.001 (Independent samples T tests). (TIFF 127 kb) [file 12862_2018_1214_MOESM1_ESM.tiff]

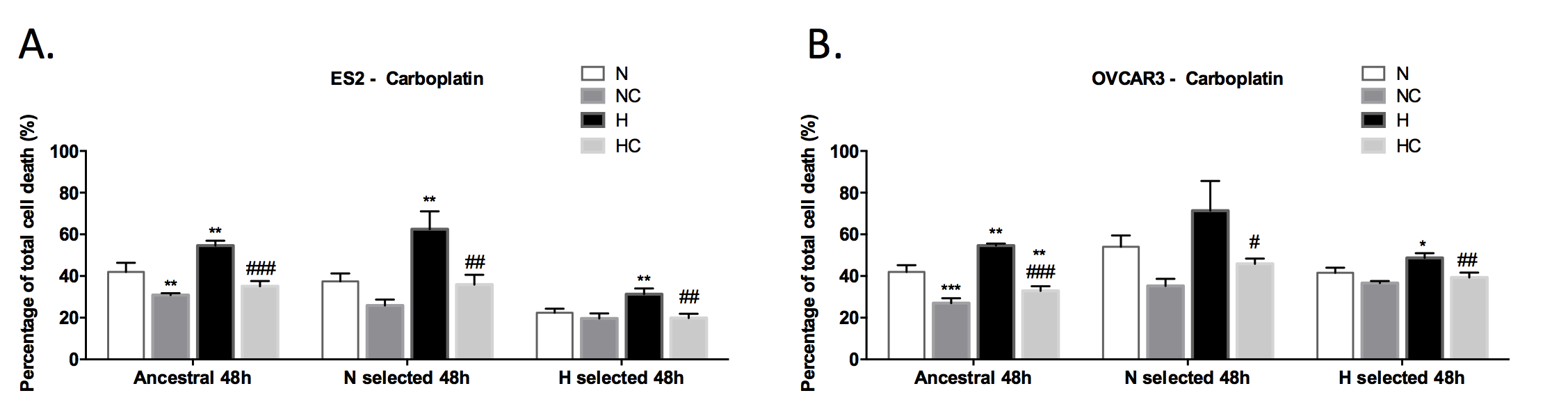

Supplement: Supplementary file 3 — Figure S2. Metabolic evolution driven by hypoxia mimicked with CoCl2 provides stronger resistance to carboplatin. Cell death levels (non-normalized values) in the presence of carboplatin for 48 h of assay for A. ES2 cells and B. OVCAR3 cells. N selected – cells selected under normoxia; H selected – cells selected under hypoxia mimicked with CoCl2; N – Normoxia; NC – Normoxia supplemented with cysteine; H – Hypoxia mimicked with CoCl2; HC – Hypoxia mimicked with CoCl2 supplemented with cysteine. Results are shown as mean ± SD. Asterisks represent statistical significance compared to cells cultured under normoxia within each cell line. Cardinals represent statistical significance compared to cells cultured under hypoxia within each cell line. *p < 0.05, **p < 0.01, ***p < 0.001 or #p < 0.05, ##p < 0.01, ###p < 0.001 (One-way ANOVA with post hoc Tukey tests). (TIFF 159 kb) [file 12862_2018_1214_MOESM3_ESM.tiff]

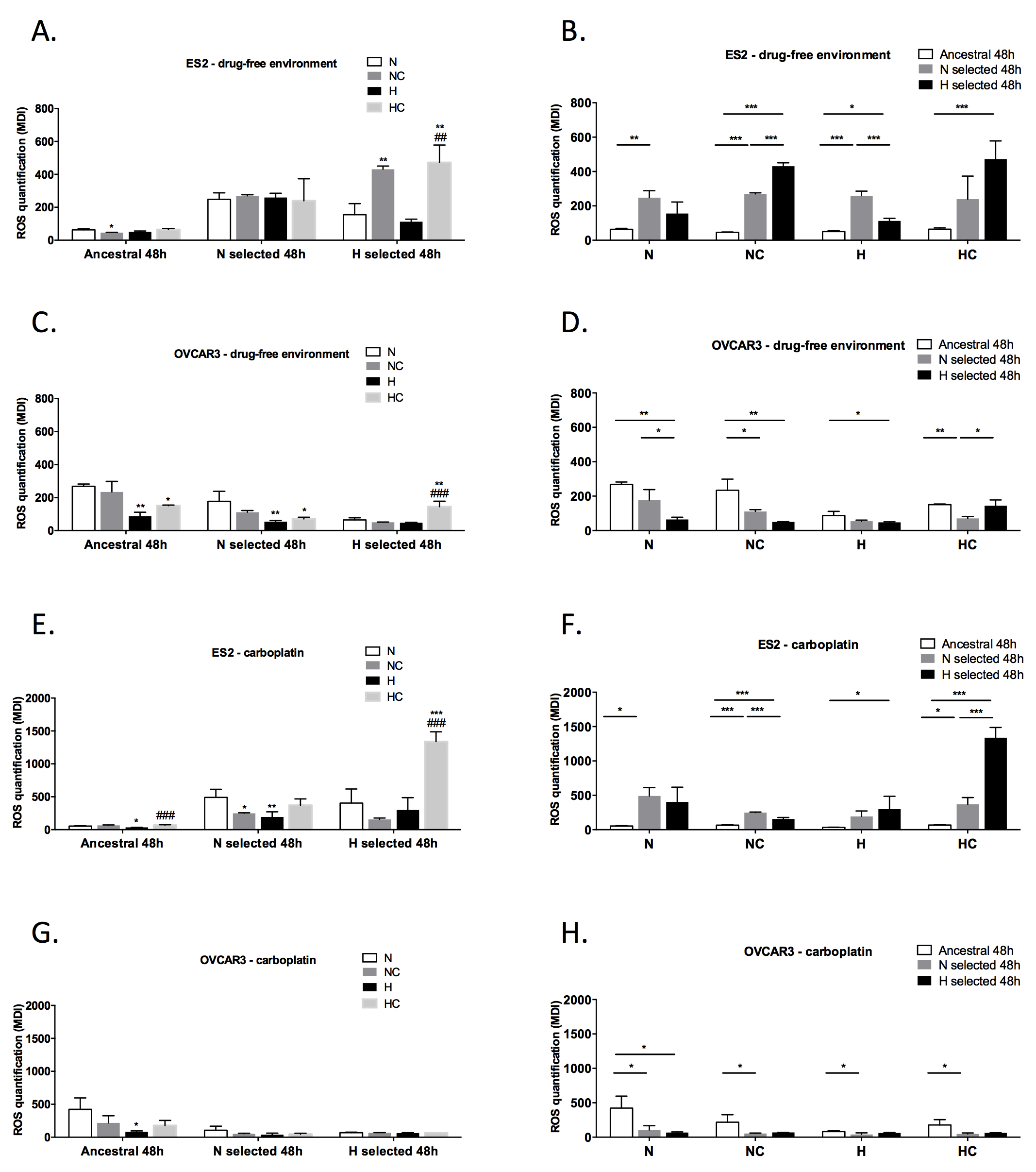

Supplement: Supplementary file 4 — Figure S3. ROS levels in ES2 (OCCC) and OVCAR3 (OSC) ancestral cells, cells selected under normoxia and under hypoxia mimicked with CoCl2. ROS levels in a drug-free environment for 48 h of assay for A. and B. ES2 cells and C. and D. OVCAR3 cells and ROS levels in the presence of Carboplatin for 48 h of assay for E. and F. ES2 cells and G. and H. OVCAR3. N selected – cells selected under normoxia; H selected – cells selected under hypoxia mimicked with CoCl2; N – Normoxia; NC – Normoxia supplemented with cysteine; H – Hypoxia mimicked with CoCl2; HC – Hypoxia mimicked with CoCl2 supplemented with cysteine. Results are shown as mean ± SD. Asterisks represent statistical significance compared to cells cultured under normoxia within each cell line. Cardinals represent statistical significance compared to cells cultured under hypoxia mimicked with CoCl2 within each cell line. *p < 0.05, **p < 0.01, ***p < 0.001 or #p < 0.05, ##p < 0.01, ###p < 0.001 (One-way ANOVA with post hoc Tukey tests). (TIFF 484 kb) [file 12862_2018_1214_MOESM4_ESM.tiff]
