# Supplementary material for: Cysteine boosters the evolutionary adaptation to CoCl2 mimicked hypoxia conditions, favouring carboplatin resistance in ovarian cancer
Source: BMC Evol Biol. 2018 Jun 19;18:97. doi: 10.1186/s12862-018-1214-1 (PMC6011206; doi:10.1186/s12862-018-1214-1)
Supplement: Supplementary file 2 — Table S1. ES2 and OVCAR3 cells resistance to hypoxia mimicked with CoCl2. Table S2. Metabolic evolution driven by hypoxia mimicked with CoCl2 provides stronger resistance to carboplatin. Table S3. ROS levels in ES2 (OCCC) and OVCAR3 (OSC) ancestral cells, cells selected under normoxia and under hypoxia mimicked with CoCl2. (DOCX 16 kb) [file 12862_2018_1214_MOESM2_ESM.docx]

**Supplementary Table I.** ES2 and OVCAR3 cells resistance to hypoxia mimicked with CoCl_2_

| Treatments – cell death analysis (48h) | Independent samples T test sig. |
| --- | --- |
| ES2-AH vs OVCAR3-AH | 0.009 |
| ES2-NH vs OVCAR3-NH | 0.009 |
| ES2-HH vs OVCAR3-HH | 0.004 |

**Supplementary Table II.** Metabolic evolution driven by hypoxia mimicked with CoCl_2_ provides stronger resistance to carboplatin

A.

| Treatments – cell death analysis (48h) | Tukey test sig. |
| --- | --- |
| ES2-AN vs ES2-ANC | 0.006 |
| ES2-AN vs ES2-AH | 0.003 |
| ES2-ANC vs ES2-AH | 0.000 |
| ES2-AH vs ES2-AHC | 0.000 |
| ES2-NN vs ES2-NH | 0.002 |
| ES2-NNC vs ES2-NH | 0.000 |
| ES2-NHC vs ES2-NH | 0.001 |
| ES2-HN vs ES2-HH | 0.006 |
| ES2-HNC vs ES2-HH | 0.001 |
| ES2-HHC vs ES2-HH | 0.001 |

B.

| Treatments – cell death analysis (48h) | Tukey test sig. |
| --- | --- |
| OVCAR3-AN vs OVCAR3-ANC | 0.000 |
| OVCAR3-AN vs OVCAR3-AH | 0.001 |
| OVCAR3-AN vs OVCAR3-AHC | 0.006 |
| OVCAR3-AH vs OVCAR3-AHC | 0.000 |
| OVCAR3-ANC vs OVCAR3-AH | 0.000 |
| OVCAR3-NNC vs OVCAR3-NH | 0.002 |
| OVCAR3-NHC vs OVCAR3-NH | 0.018 |
| OVCAR3-HN vs OVCAR3-HH | 0.013 |
| OVCAR3-HNC vs OVCAR3-HH | 0.000 |
| OVCAR3-HHC vs OVCAR3-HH | 0.003 |

**Supplementary Table III.** ROS levels in ES2 (OCCC) and OVCAR3 (OSC) ancestral cells, cells selected under normoxia and under hypoxia mimicked with CoCl_2_

A.

| Treatments – ROS quantification (48h) | Tukey test sig. |
| --- | --- |
| ES2-AN vs ES2-ANC | 0.021 |
| ES2-AHC vs ES2-ANC | 0.016 |
| ES2-HN vs ES2-HNC | 0.003 |
| ES2-HN vs ES2-HHC | 0.001 |
| ES2-HH vs ES2-HNC | 0.001 |
| ES2-HH vs ES2-HHC | 0.001 |

B.

| Treatments – ROS quantification (48h) | Tukey test sig. |
| --- | --- |
| ES2-AN vs ES2-NN | 0.006 |
| ES2-ANC vs ES2-NNC | 0.000 |
| ES2-ANC vs ES2-HNC | 0.000 |
| ES2-HNC vs ES2-NNC | 0.000 |
| ES2-AH vs ES2-NH | 0.000 |
| ES2-AH vs ES2-HH | 0.013 |
| ES2-HH vs ES2-NH | 0.000 |
| ES2-AHC vs ES2-HHC | 0.006 |

C.

| Treatments – ROS quantification (48h) | Tukey test sig. |
| --- | --- |
| OVCAR3-AN vs OVCAR3-AH | 0.001 |
| OVCAR3-ANC vs OVCAR3-AH | 0.004 |
| OVCAR3-AN vs OVCAR3-AHC | 0.015 |
| OVCAR3-NN vs OVCAR3-NH | 0.006 |
| OVCAR3-NN vs OVCAR3-NHC | 0.013 |
| OVCAR3-HN vs OVCAR3-HHC | 0.003 |
| OVCAR3-HNC vs OVCAR3-HHC | 0.001 |
| OVCAR3-HH vs OVCAR3-HHC | 0.001 |

D.

| Treatments – ROS quantification (48h) | Tukey test sig. |
| --- | --- |
| OVCAR3-AN vs OVCAR3-HN | 0.001 |
| OVCAR3-NN vs OVCAR3-HN | 0.021 |
| OVCAR3-ANC vs OVCAR3-NNC | 0.017 |
| OVCAR3-ANC vs OVCAR3-HNC | 0.002 |
| OVCAR3-AH vs OVCAR3-HH | 0.035 |
| OVCAR3-AHC vs OVCAR3-NHC | 0.007 |
| OVCAR3-HHC vs OVCAR3-NHC | 0.01 |

E.

| Treatments – ROS quantification (48h) | Tukey test sig. |
| --- | --- |
| ES2-AN vs ES2-AH | 0.015 |
| ES2-ANC vs ES2-AH | 0.001 |
| ES2-AHC vs ES2-AH | 0.000 |
| ES2-NN vs ES2-NNC | 0.026 |
| ES2-NN vs ES2-NH | 0.008 |
| ES2-HN vs ES2-HHC | 0.000 |
| ES2-HH vs ES2-HHC | 0.000 |
| ES2-HNC vs ES2-HHC | 0.000 |

F.

| Treatments – ROS quantification (48h) | Tukey test sig. |
| --- | --- |
| ES2-AN vs ES2-NN | 0.022 |
| ES2-ANC vs ES2-NNC | 0.000 |
| ES2-ANC vs ES2-HNC | 0.000 |
| ES2-NNC vs ES2-HNC | 0.000 |
| ES2-AH vs ES2-HH | 0.026 |
| ES2-AHC vs ES2-NHC | 0.019 |
| ES2-AHC vs ES2-HHC | 0.000 |
| ES2-NHC vs ES2-HHC | 0.000 |

G.

| Treatments – ROS quantification (48h) | Tukey test sig. |
| --- | --- |
| OVCAR3-AN vs OVCAR3-AH | 0.022 |

H.

| Treatments – ROS quantification (48h) | Tukey test sig. |
| --- | --- |
| OVCAR3-AN vs OVCAR3-NN | 0.025 |
| OVCAR3-AN vs OVCAR3-HN | 0.016 |
| OVCAR3-ANC vs OVCAR3-NNC | 0.044 |
| OVCAR3-AH vs OVCAR3-NH | 0.043 |
| OVCAR3-AHC vs OVCAR3-NHC | 0.03 |
